# Tracking Down Abstract Linguistic Meaning: Neural Correlates of Spatial Frame of Reference Ambiguities in Language

**DOI:** 10.1371/journal.pone.0030657

**Published:** 2012-02-17

**Authors:** Gabriele Janzen, Daniel B. M. Haun, Stephen C. Levinson

**Affiliations:** 1 Behavioural Science Institute, Radboud University Nijmegen, Nijmegen, The Netherlands; 2 Donders Institute for Brain, Cognition and Behaviour, Radboud University Nijmegen, Nijmegen, The Netherlands; 3 Max Planck Institute for Psycholinguistics, Nijmegen, The Netherlands; 4 Max Planck Institute for Evolutionary Anthropology, Leipzig, Germany; City of Hope National Medical Center and Beckman Research Institute, United States of America

## Abstract

This functional magnetic resonance imaging (fMRI) study investigates a crucial parameter in spatial description, namely variants in the frame of reference chosen. Two frames of reference are available in European languages for the description of small-scale assemblages, namely the intrinsic (or object-oriented) frame and the relative (or egocentric) frame. We showed participants a sentence such as “the ball is in front of the man”, ambiguous between the two frames, and then a picture of a scene with a ball and a man – participants had to respond by indicating whether the picture did or did not match the sentence. There were two blocks, in which we induced each frame of reference by feedback. Thus for the crucial test items, participants saw exactly the same sentence and the same picture but now from one perspective, now the other. Using this method, we were able to precisely pinpoint the pattern of neural activation associated with each linguistic interpretation of the ambiguity, while holding the perceptual stimuli constant. Increased brain activity in bilateral parahippocampal gyrus was associated with the intrinsic frame of reference whereas increased activity in the right superior frontal gyrus and in the parietal lobe was observed for the relative frame of reference. The study is among the few to show a distinctive pattern of neural activation for an abstract yet specific semantic parameter in language. It shows with special clarity the nature of the neural substrate supporting each frame of spatial reference.

## Introduction

The aim of the study reported here is to shed light on the cortical systems underlying the spatial frame of reference concepts crucially involved in spatial language. Here we use linguistic ambiguities of frames of reference to pinpoint the cortical activations associated with each of two distinct frames of reference. In doing so, we are able to localize the circuitry associated with an abstract parameter of linguistic meaning.

**Figure 1:**
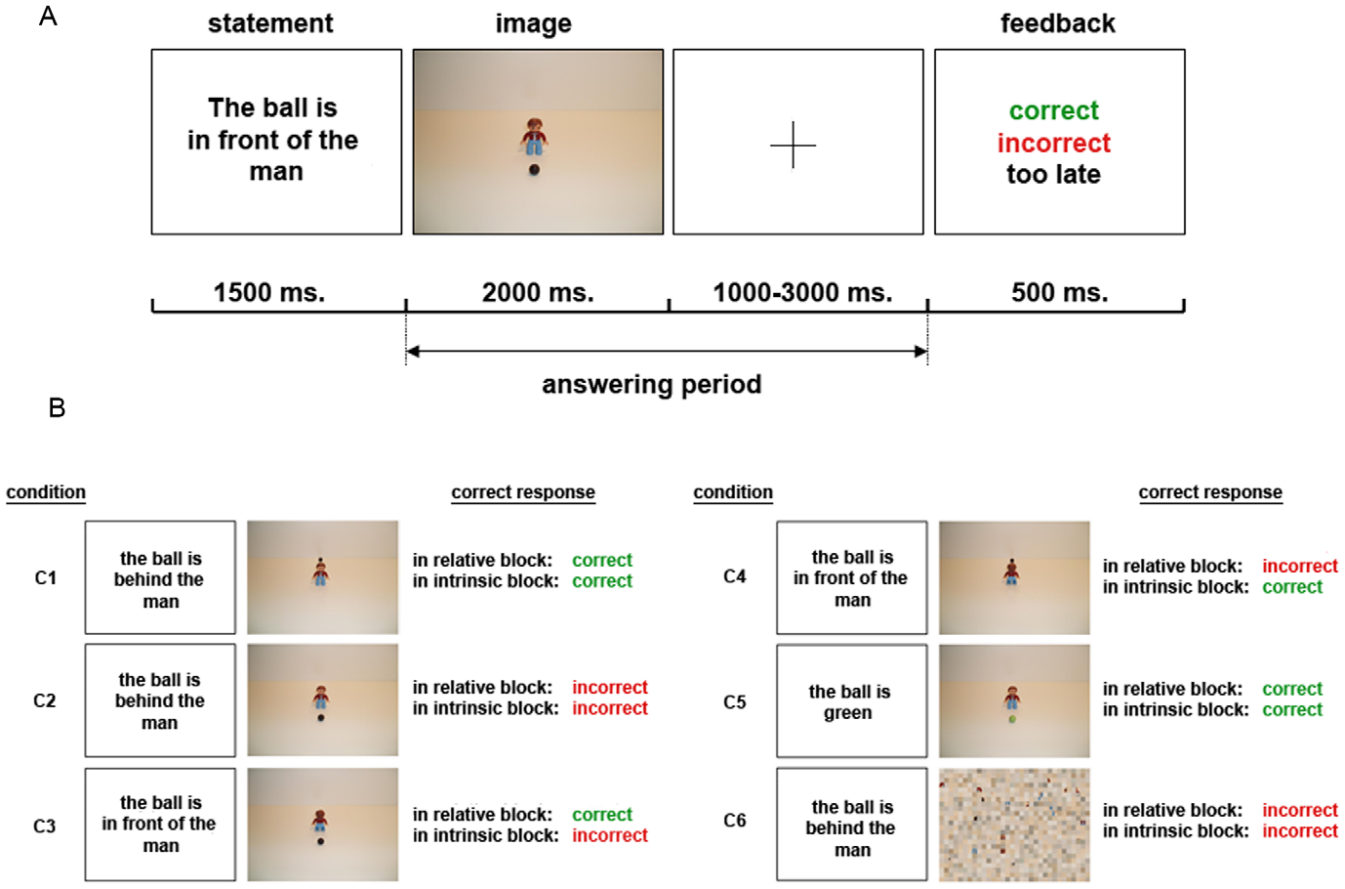
Sentence-picture matching task and conditions. (A) Participants saw a sentence for 1500 ms and afterwards a scene for 2000 ms. Immediately thereafter a fixation cross with a variable time interval from 1000–3000 ms followed during which participants responded. Afterwards feedback was displayed for 500 ms. (B) Stimuli from all conditions were presented randomly intermixed within the two blocks of relative and intrinsic feedback.

Language constructs ambiguities – two takes on the same linguistic string. For example, *Visiting relatives can be boring* could mean ‘going to see relatives is boring’, or it could mean ‘relatives who come and see us are boring’. In a similar way, *The ball is in front of the man* could mean ‘the ball is at the man's front’ (see Figure 1, Panel A) or ‘the ball is between me and the man’. In some spatial arrangements, both kinds of interpretation may be equally valid (see e.g. Panel B, C1). In the latter case, how could we tell which way an observer is thinking? We will suggest that each perspective has a distinctive neural signature.

Ambiguities in language are similar to ambiguities elsewhere in perception. For example, a wire-framed image like a Necker cube can be perceived as if from two different locations, a high vantage point or a low vantage point, requiring a reversal of the depth of the implied ‘faces’. The reversal can be induced by rotating the wire frame on a monitor [1], and the time-course and location of activation associated with reversal measured by EEG and fMRI respectively. Spontaneous reversals occur early on in stimulus presentation and are associated with specific neural signatures [2]. It has also been suggested that there is a specific signature of conscious awareness of multistable images [3].

Language is not vision, and there is no corresponding evidence for spontaneous reversals. Nevertheless, the processing of ambiguities shows special neural activations, for example, when the less frequent lexical meaning is contextually required [4]. Here we focus not on the processing of the ambiguity itself, but rather on the methodological trick ambiguities afford – namely the possibility of using a sustained ambiguity to explore the activation of a single point change in interpretation. Holding the objective perceptual stimulus constant, we can explore the neural correlates of a subjective change in meaning.

Why is this interesting? Because hitherto the neurocognition of meaning has for the most part lacked the necessary degree of precision with respect to different possible aspects of meaning. Many studies have examined what areas are activated in a semantic vs. a syntactic task [5], and have built on contrastive electrophysiological signals of semantic vs. syntactic violations [6]. But relatively few studies have been able to isolate very precise aspects of meaning and their corresponding neurocognitive signatures, with three kinds of exception. The first, following up the lesion literature [7] concerns the possible category-specific nature of the neurocognitive processing of words (see [8] for a review of imaging studies). Categories such as tools vs. animals, as explored through corresponding words, seem to show different patterns of neural activation. However, the activation patterns associated with such domains are never very precise, and may in any case be due to non-linguistic downstream activation of, e.g., visual properties. A second line of work has explored more high-level categories or domains, like the difference between spatial relations as encoded in prepositions vs. object names [9]–[11], finding greater activation of parietal and frontal areas for the spatial relations (see also [12], [13] for an overview on imaging studies on spatial semantics).

The third area, where localizations of greater precision have been made, concerns action words that involve specific body parts (such as *kick*, or *punch*), where the relevant parts of motor and pre-motor cortex may be activated in a somatotopic manner [14], [15]: thus *kick* activates a part of the motor strip close to the vertex, an area associated with the leg, but *punch* a dorso-lateral part, associated with the arm. These data suggest that the meaning of words cannot reside in a single cortical locus, as used to be thought on the basis of lesion data, but rather invoke distributed circuitry activating areas specially connected to their referents. Of course these action words, in their reference to concrete body-parts, are prime candidates for any such “embodied” representation of meaning. “It remains to be determined”, says Pulvermüller [15], “whether it might be possible to read aspects of meaning of other words, such as abstract terms, from the cortex in a similar manner”.

The study reported here picks up this challenge. We show here that it is possible to pinpoint the contribution of specific patterns of neural activation to an abstract aspect of meaning, namely the specific frame of reference or spatial coordinate system associated with the description of a spatial scene.

In language three major types of reference systems can be distinguished [16]: intrinsic (object-oriented), relative (egocentric) and absolute (world-oriented). Most European languages predominantly use a relative frame of reference with terms like ‘front’, ‘back’, ‘left’ and ‘right’ to form descriptions such as: “The ball is in front of the man (from my point of view)”. But the same languages can also use an intrinsic frame of reference, a coordinate system making use of the named facts of a reference object as in “at the back of the house”. Some other languages around the world predominantly use a third system, a so-called *absolute* frame of reference, in which linguistic descriptions use cardinal-direction type systems comparable to our North-South-East-West. [17]–[19]. The preferred frame of reference within a language may influence the way an environment is cognitively represented [16], [20]. Here we use the term ‘intrinsic’ for an object-centered reference frame and the term ‘relative’ for a viewer-centered frame [16].

An implicit sentence picture-matching task was used to investigate differential neural correlates for relative and intrinsic frames of reference (see Figure 1). Participants read a sentence describing a spatial scene, followed by viewing a picture, and decided whether the sentence matched the picture or not. During a block of trials, consistent feedback (correct, incorrect) was given so inducing either a relative or intrinsic frame. Midway through the trials, the second block began and the feedback was switched to the alternative reference frame without further explanation (see Methods and Materials section below).

## Results

### Behavioral data

The mean number of errors during the intrinsic and relative block was not significantly different (total intrinsic frame of reference: 11.68; total relative frame of reference: 12.71). Also, no difference in the mean number of errors was found between the specific pictures under each interpretative frame, i.e. between the condition pairs intrinsic C1 and relative C1 (2.18 and 2.39) (see Figure 1B for examples), intrinsic C2 and relative C2 (1.25 and 1.07), intrinsic C3 and relative C4 (4.21 and 4.39), intrinsic C4 and relative C3 (2.75 and 3.68), intrinsic C5 and relative C5 (1.11 and 0.89), and intrinsic C6 and relative C6 (0.18 and 0.29). However, the mean amount of errors during the first block was significantly lower than during the second block (8.75 and 15.64; *t*(27) = −3.63, *p*<0.001) – not unexpected, since the conditions changed without warning. No significantly different mean number of errors was found between the 2^nd^ block in the intrinsic frame of reference mode and the 2^nd^ block in the relative frame of reference mode (12.86 and 18.43).

The mean response times for the two frames of reference showed a trend towards significance (*t*(27) = −1.87, *p* = 0.07): Response times were marginally faster for the relative frame of reference (854 ms) than for the intrinsic frame of reference (886 ms). The mean response times for the first block and the second block showed no difference (respectively 868 ms and 873 ms). Also, no differences in mean response times were found between the pictures under distinct frames, i.e. intrinsic C2 and relative C2 (1010 ms and 1000 ms; see Figure 1B), intrinsic C5 and relative C5 (801 ms and 795 ms), and intrinsic C6 and relative C6 (649 ms and 663 ms). Mean response-time differences were found between the condition pairs intrinsic C1 and relative C1 (946 ms and 885 ms; *t*(27) = 2.46, *p*<0.05), and intrinsic C3 and relative C4 (1009 ms and 948 ms; *t*(27) = 2.08, *p*<0.05). The mean response times of conditions intrinsic C4 and relative C3 showed a trend towards faster relative responses (903 ms and 834 ms; *t*(27) = 1.85, *p* = 0.08).

### FMRI data

Compared to the low level baseline (scrambled pictures, C6 in Figure 1B), relative, and intrinsic trials strongly activated bilateral occipitotemporal cortices. This region is usually referred to as the ventral visual pathway [21].

### Neural correlates of intrinsic and relative frames of reference

**Figure 2:**
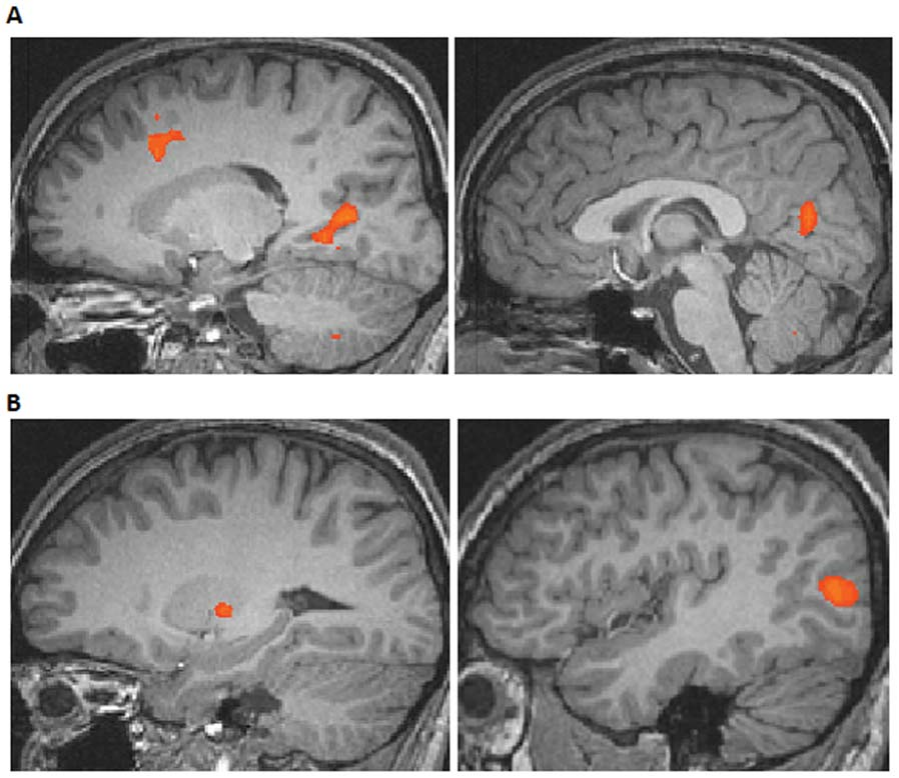
Brain areas showing increased responses for intrinsic sentences versus relative sentences. (A) Increased activity in the right posterior cingulate and the superior frontal gyrus. (B) Increased activity in the left occipital gyrus and the putamen.

First of all we analyzed the sentence part of the trials. All sentences given in the intrinsic block as compared to all sentences given in the relative block revealed increased activity in the right posterior cingulate gyrus, the fusiform gyrus, the superior frontal gyrus (Figure 2A, Table 1), as well as in the left inferior occipital gyrus, in the putamen, the supramarginal gyrus, and the parahippocampal gyrus (Figure 2B, Table 1). Relative sentences as compared to intrinsic sentences showed no increased activity at the threshold of FDR (false discovery rate) p<0.05. Relative sentences as compared to intrinsic sentences showed an increase in activity in the left superior frontal gyrus (x = −8, y = 68, z = 13, BA 10) at a higher threshold only (p<0.003).

**Table 1:** Increased brain activity for intrinsic sentences vs. relative sentences.

	Talairach coordinates					
Anatomical region	*x*	*y*	*z*	BA	Size (mm^3^)	Peak *T* value
Right						
Posterior cingulate gyrus	1	−68	13	30	990	6.76
Posterior cingulate gyrus	12	−61	8	30	1715	6.77
Posterior cingulate gyrus	12	−47	22	31	69	5.75
Fusiform gyrus	27	−58	−12	37	233	5.0
Putamen	18	16	6		17	4.8
Superior frontal gyrus	16	17	39	8	281	5.57
Left						
Inferior occipital gyurs	−43	−76	7	19	1057	5.70
Putamen	−28	−14	4		171	5.6
Supramarginal gyrus	−50	−22	19	40	84	5.2
Parahippocampal gyrus	−15	−33	−5	30	20	5.1

*P*<0.0001 (FDR<0.05).

Secondly we analyzed the picture part of the trials. Intrinsic pictures (C1–C4) as compared to high level baseline trials (C5) revealed increased activity in the temporal and occipital gyrus (Table 2). Relative pictures (C1–C4) as compared to high level baseline trials (C5) revealed a widespread network of activity. Increased activity was observed in the left parietal lobe, in bilateral frontal areas, and in the precentral gyrus (Table 3).

**Table 2:** Increased brain activity for intrinsic conditions C1–C4 vs. intrinsic baseline C5.

	Talairach coordinates					
Anatomical region	*x*	*y*	*z*	BA	Size (mm^3^)	Peak *T* value
Left						
parahippocampal gyrus	−32	−31	−15	36	64	4.55
middle occipital gyrus	−28	−83	8	18	237	4.88

*P*<0.001 (cluster-size corrected).

**Table 3:** Increased brain activity for relative conditions C1–C4 vs. relative baseline C5.

	Talairach coordinates					
Anatomical region	*x*	*y*	*z*	BA	Size (mm^3^)	Peak *T* value
Right						
Superior frontal gyrus	9	65	9	10	75	4.84
Left						
Superior parietal lobule	−30	−50	30	7	40	4.53
Medial aspect of frontal lobe	−6	4	51	6	180	4.68
Precentral gyrus	−45	−1	47	6	382	4.55
Superior frontal gyrus	−14	−1	62	6	335	4.79

*P*<0.001 (cluster-size corrected).

**Figure 3:**
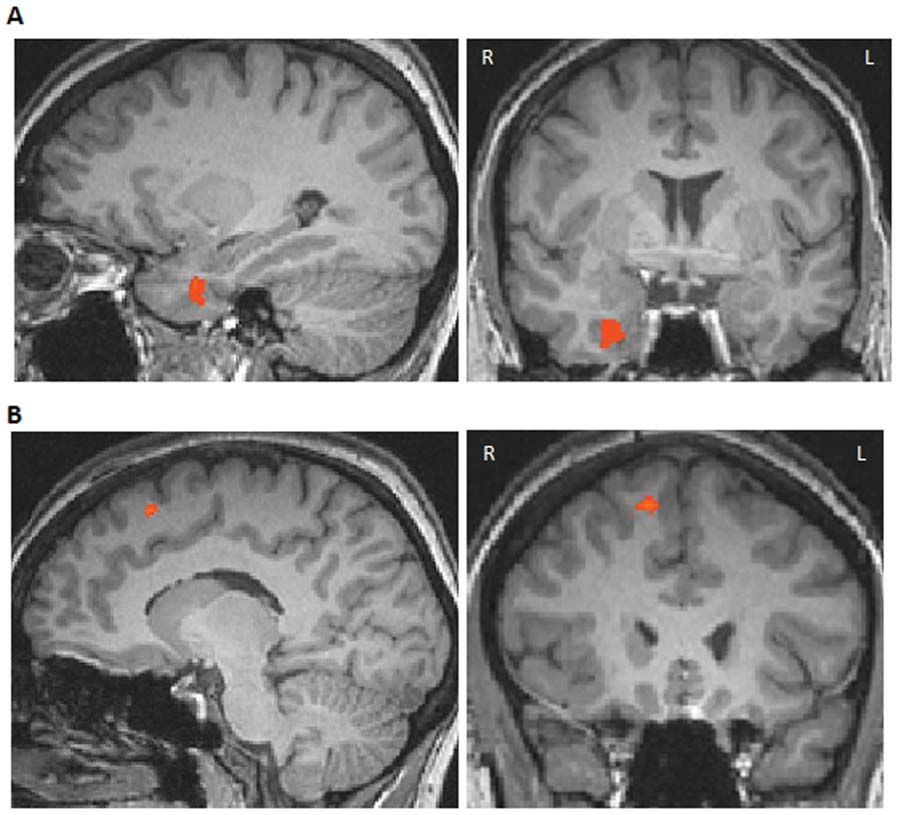
Brain areas showing increased responses for intrinsic and relative pictures. (A) Increased activity in the right parahippocampal gyrus for intrinsic pictures compared to relative pictures. (B) Increased activity in the right superior frontal gyrus for relative pictures compared to intrinsic pictures.

We focused on the direct comparison between relative and intrinsic pictures. The direct comparison of all experimental conditions (C1–C4) from the intrinsic block with the same conditions (C1–C4) from the relative block revealed increased activity in the right parahippocampal gyrus for the intrinsic conditions (x = 25, y = 0, z = −26, 239 mm^3^ BA 38 , Peak t value 4.48, see Figure 3A). Besides the parahippocampal activity, an increase in activity was detected in the right superior occipital gyrus (x = 25, y = −69, z = 14, 40 mm^3^, BA 19, Peak t value 4.44).

The same conditions when comparing relative with intrinsic pictures revealed increased activity in the right superior frontal gyrus (x = 10, y = 20, z = 52, 72 mm^3^, BA 6, Peak t value 4.98, Figure 3B).

When comparing only the C1 and C2 conditions from the intrinsic with the relative block – conditions in which the correct responses were identical for both the relative as well as the intrinsic frame of reference – strongly increased activity was found for the intrinsic conditions in the left temporal pole (x = −41, y = 7, z = −26, 271 mm^3^, BA 38, Peak t value 4.11 ) as well as in the right temporal pole (x = 24y = 4, z = −32, 57 mm^3^, BA 38, Peak t value 3.5 ) and in the bilateral parahippocampal gyrus (left: x = −33, y = 0, z = −30, 46 mm^3^, BA 36, Peak t value 4.0; right: x = 26, y = −1, z = 26, BA 36, 138 mm^3^, Peak t value 4.12 ). The relative as compared to the intrinsic C1 and C2 conditions revealed increased activity for the relative trials in the superior frontal gyrus (x = 15, y = −13, z = 67, BA 6) at a higher threshold only (*p*<0.003, 38 mm^3^). No activity was observed at the lower threshold (*p*<0.001).

## Discussion

In the present event-related fMRI study we investigated the cortical systems underlying the concepts of spatial frames of reference that are crucially involved in spatial language. Using ambiguous sentences that could be interpreted in either frame, and inducing one or the other frame in a verification task, we have been able to pinpoint distinct neural correlates for an abstract semantic parameter in language, namely the frame of reference associated with the interpretation of an ambiguous spatial scene. In the following we discuss our results in detail.

Participants performed a sentence picture-matching task and received feedback supporting either an intrinsic or a relative frame of reference, depending on which one of the two blocks they were in. Each trial consisted of a sentence and a picture, which were analyzed in separate contrasts. Comparing all intrinsic sentences with the same number of identical sentences given in the relative feedback block revealed a widespread network of increased occipital, frontal, and temporal regions for the intrinsic sentences (Table 1, Figure 2). No increased activity was observed for the relative sentences. Firstly, this result shows that the differentiation of intrinsic and relative reference frames starts early already at the level of sentence processing. Secondly, the increased neural network for sentences in the intrinsic feedback block might reflect the larger effort that participants need to interpret the sentences intrinsically. This could be due to the native language of the participants (Dutch) which is a language that dominantly makes use of the relative reference frame [16], [22]. This is further supported by findings showing the posterior cingulate cortex involved in task engagement and change detection [23], and the supramarginal gyrus involved in language processing more generally [24] (Table 1). Thirdly and interestingly, intrinsic sentences already engage the parahippocampal gyrus, a brain region also involved when processing the intrinsic pictures. This shows that the sentences are already fully processed intrinsically and might enable the participants to simply map the picture following the sentence on the intrinsic representation.

Intrinsic pictures compared directly to relative pictures from all experimental conditions showed increased activity in the right parahippocampal gyrus and the superior occipital gyrus (Figure 3A). Relative trials as compared to intrinsic trials on the other hand revealed increased neural activity in the frontal lobe (Figure 3B). Further evidence for these different neural correlates for both frames of reference comes from contrasting the C1 and C2 conditions only. In these conditions both frames of references are always correct or incorrect in both feedback blocks. The results show bilateral parahippocampal activity for intrinsic pictures and the superior frontal gyrus for relative pictures.

The behavioral results showed no difference in error rates between intrinsic and relative frames of reference. Therefore the fMRI results that include correct responses only were not biased by differences in errors between both frames of reference. In the second block participants made significantly more errors than in the first block due to shifting to the alternative frame of reference. The response times showed that relative responses are marginally faster than intrinsic responses. This trend most likely reflects the dominance of the relative reference frame in most speakers of Dutch, the language of the participants [16], [22]. The response times are modeled as parametric modulation regressors in the fMRI analyses, which rules out that the differences in neural correlates are due to or influenced by the marginally faster responses in the relative block.

Coding space within different frames of reference can require different cognitive processes [25]–[27]. FMRI data have suggested that different frames of reference can be linked to differential patterns of neural activation [28]–[30]. It has been proposed that the hippocampus is involved in the creation of absolute representations [31], [32], while parietal lobes have been argued to subserve especially relative spatial computations [33]. Recent results show shared activity in occipital, superior parietal, superior frontal and left inferior temporal brain regions in both frames [34].

Comparing trials with intrinsic as well as relative pictures to baseline trials (Table 2 and 3) we found a shared widespread network with increased activity in occipital, parietal, temporal and frontal brain regions. This is in line with evidence from an fMRI study that distinguished viewer-, object-, and landmark-centered distance judgments, and found common activity for all three types in bilateral parietal, occipital, and right frontal premotor regions as well [29]. These results provide evidence for a widespread network activated when any of the different frames of references are used to make a decision.

The present study used a sentence-picture matching task within two unmarked blocks, with the relative or intrinsic frame cued only by feedback about correct/incorrect matches. The task instructions required participants simply to decide whether a sentence matched the following picture and remained identical for both blocks with intrinsic and relative feedback. This allowed us to use exactly the same stimulus material under two variants to indirectly induce interpretations, without explicit instruction about reference frames. Therefore the distinct neural networks observed for the relative and the intrinsic frame of reference were not influenced by differences in stimuli or by different interpretations of complex task instructions. This method offers a distinct advantage over previous studies that were unable to use identical stimuli to investigate the different reference frames and needed to inform participants directly about the explicit reference frame that should be used to solve a task [29], [34]. Identical stimuli for both frames of reference allowed us to analyze the neural correlates of a change solely in the subjective meaning or interpretation of the sentences. This is especially true for the conditions C1 and C2 (Figure 1B), stimuli that are ambiguous between the intrinsic and the relative frame of reference, allowing both interpretations as correct or incorrect solutions. We were able to observe distinct neural correlates for each frame - the intrinsic reference frame in the temporal lobe and for the relative reference frame in the frontal lobe - when directly contrasting these trials from both feedback blocks.

Numerous recent neuroimaging studies have revealed brain areas involved in spatial processing. Special foci have been the temporal and the parietal lobe [35]. There is much evidence from animal as well as human studies for the involvement of the mediotemporal lobe, including the hippocampal formation and the parahippocampal region, in navigation and the representation of space [36], [37]. In the rat hippocampus specific neurons called place cells encode the animal's location [31]. This was taken as support for the existence of an allocentric representation of space or cognitive map, using world-centered coordinates in the hippocampus. Recently place cells were found in the human hippocampus while participants navigated through a virtual maze [32].

In the present study we directly compared intrinsic with relative trials and observed increased activity for intrinsic trials in bilateral parahippocampal gyrus (Figure 3), an area closely connected to the hippocampus through the entorhinal and perirhinal gyrus. Recent neuroimaging studies emphasize the importance of the parahippocampal gyrus for the recognition of familiar as well as novel spatial environments and scenes [38]–[43] and for object-location memory [36], [44], [45]. To correctly solve intrinsic trials participants needed to consider the spatial relation of two objects and decide whether the scene matched a previously presented sentence. Therefore scene representation within the parahippocampal gyrus should be able to support intrinsic frames of reference.

Whereas the right medial temporal area is associated with memory for object locations in an allocentric frame of reference, fMRI data has shown that the parietal lobe is associated with representations of object locations in an egocentric reference frame [33]–[35], [46]. The present data when comparing relative trials to baseline trials supports the involvement of the parietal lobe. We observed increased activity in the left parietal lobe for the relative frame of reference only, confirming neurophysiological studies which report the involvement of the parietal lobe in egocentric coding [33].

Relative trials as compared to intrinsic trials also showed strongly increased activity in superior frontal gyrus (Figure 3B). This is in line with findings from researchers [29] who have observed a parietal/frontal network for viewer-centered coding.

In essence, in the present fMRI study we used a sentence-picture matching task and gave feedback in two separate blocks supporting either a relative or an intrinsic frame of reference. With this method we were able to pinpoint the patterns of neural activity associated with each frame of reference, by using the exact same perceptual stimuli. Intrinsic conditions as compared to relative conditions showed increased activity in the parahippocampal gyrus whereas relative trails compared to intrinsic trials revealed increased neural activity in the frontal lobe. The present results show differential neural networks for both frames of reference that are (in most languages) crucial to spatial language. Very few earlier studies have been able to precisely identify a distinctive pattern of neural activity for a specific but abstract semantic parameter in language. Earlier studies have focused, for example, on spatial prepositions as a class [9], [10]. Just a few have gone further and explored the neural signatures of specific dimensions of meaning, for example the temporal vs. spatial meanings of prepositions [47] or the manner/path distinction in motion coding [48]. Here we have been able to identify the specific neural activation patterns involved in the two different senses of spatial relators like ‘in front of’ – either involving an object centered (intrinsic) or an egocentric (relative) frame of reference.

A final issue concerns the role of language vs. non-linguistic spatial cognition in the activation patterns we have observed. This was not directly addressed in the current experiment, which did not contrast linguistic vs. non-linguistic conditions. However, separate analyses of the brain response to the verbal part of the stimulus and the (following) pictorial part of the stimulus showed early differentiation of the two frames of reference in both modalities, with some striking similarities of activation within the frame of reference across both modalities. This frame-of-reference specialization in both linguistic and visual interpretation is perhaps not surprising since the use of language has to be supported by the requisite underlying perceptual and conceptual activations. Nevertheless, there is converging evidence from neuropsychology [49] and imaging studies [50], [11] that spatial language requires a categorical rather than the metric or coordinate spatial conception involved in action and perception, even though both systems must somehow talk to one another.

## Materials and Methods

### Participants

Twenty-eight healthy human adults (16 women, 12 men) gave informed written consent before participating in the experiment. Twenty-six participants were included in the fMRI analyses. All participants were right-handed and had normal or corrected-to-normal vision. The age ranged from 20–34 (average 24.3, Std = 3.1). The study was approved by the CMO Committee on Research Involving Human Subjects (Region Arnhem-Nijmegen).

### Materials and Procedure

Participants saw a sentence describing a spatial scene from a first-person perspective and had to decide if the following picture and the sentence matched. The timing details of the task are provided in Figure 1. Each picture always contained a ball and an object or a person or an animal, such as old or young women and men, different animals, different types of vehicles (e.g. jeep, bulldozer) and objects with a clear front and back such as a chair, piano, and cabinet (32 different objects in total). Since the ball has no intrinsic front it served as an ideal referent object in all trials. Participants were instructed to decide as accurately and as quickly as possible if the sentence and the picture match. No further instruction and information about different reference frames was given. They responded with their right hand by pressing a key with the index finger for a correct decision and a second key with the middle finger for an incorrect judgment. They received feedback after each trial that indicated that the answer was correct, incorrect or given too late.

Four experimental conditions C1–C4 were included twice in two separate blocks; relative and intrinsic. Two baseline tasks were included (see Figure 1), a high level baseline (C5) and a low level baseline (C6). In total 320 trials were shown randomly intermixed within each block. Midway through the trials the second block started and the feedback switched to the alternate reference frame. Half of the participants started with the relative block and switched to the intrinsic feedback block whereas the other half received the blocks in the reversed order. Participants were not informed about the switch in feedback. The C1 and C2 conditions included 16 trials each. The C3–C6 conditions included 32 trails each. The C1 and C2 conditions included less trials then the other conditions since the correct and incorrect answers of these trials were identical for both the relative and the intrinsic block. The feedback is meaningless in these trials for learning the switch in reference frames, and therefore the lower number of trials in the conditions allowed a faster learning of the switch from one reference frame block to the other.

### fMRI procedure

A 3 Tesla MRI system (Siemens TRIO, Erlangen, Germany) was used to acquire functional images of the whole brain. Using a gradient-echo-planar scanning (EPI) sequence 36 axial slices per functional volume were obtained for each participant (voxel-size 3×3×3 mm, TR = 2270 ms, field of view = 192, TE = 30 ms, flip angle = 75). All functional images were acquired in one run that lasted for 50 minutes. Following the acquisition of functional images a high-resolution anatomical scan (T1-weighted MP-RAGE, 176 slices) was acquired.

### fMRI data analysis

FMRI data were preprocessed and analyzed using BrainVoyager QX (Brain Innovation, Maastricht, The Netherlands). The first five volumes of the EPI data per participant were discarded to allow for T1 equilibration. Functional images were corrected for motion and slice scan time acquisition. Data were temporally smoothed with a high pass filter removing frequencies below 3 cycles per time course. Functional images were coregistered with the anatomical scan and transformed into Talairach coordinate space using the 9-parameter landmark method of Talairach and Tournoux [51]. Images were spatially smoothed with a FWHM Gaussian kernel of 6 mm.

Statistical analyses were performed in the context of the general linear model. We included 14 regressors of interests based on the experimental conditions. These conditions included two regressors for the sentences, one modeling all intrinsic sentences and one modeling all relative sentences, and 12 regressors for the picture trials (see Figure 1). Additionally two regressors of no interest for the error trials were included modeling intrinsic and relative error trials separately. Participants response times were included as parametric modulation regressors to take into account the marginal faster responses for relative trials. This rules out response times as a nuisance covariate and excludes that any difference in responses times possibly resulting from differences in attentional demands provides the basis for the results. For the analysis of the sentence part all trials were included, for analyses of the picture part only correct responses were included in the reported analyses. Event-related hemodynamic responses for each of the different event-types were modeled as delta functions convolved with a synthetic hemodynamic response function lasting 1500 ms for the sentences and 2000 ms for the picture trials. Random-effects whole brain group analyses were performed. Specific effects were tested by applying linear contrasts to the parameter estimates for each event as obtained in the random effects group analyses. The statistical threshold for the group analyses was set at P<0.05, false discovery rate (FDR) corrected for multiple comparisons [52], and at an exploratory lower threshold at *p*<0.001 at the voxel level with a minimum cluster size of 40 mm^3^, uncorrected for multiple comparisons [53].
